# Graphene/Heterojunction Composite Prepared by Carbon Thermal Reduction as a Sulfur Host for Lithium-Sulfur Batteries

**DOI:** 10.3390/ma16144956

**Published:** 2023-07-12

**Authors:** Jiahao Li, Bo Gao, Zeyuan Shi, Jiayang Chen, Haiyang Fu, Zhuang Liu

**Affiliations:** Key Laboratory for Ecological Metallurgy of Multimetallic Mineral, Ministry of Education, Northeastern University, Shenyang 110819, China; surflijh@163.com (J.L.); surfshizy@163.com (Z.S.); neuchenjy@163.com (J.C.); surffuhy@163.com (H.F.); surfliuz@163.com (Z.L.)

**Keywords:** graphene, nanocomposite, heterojunction, lithium-sulfur battery

## Abstract

An interlayer nanocomposite (CC@rGO) consisting of a graphene heterojunction with CoO and Co_9_S_8_ was prepared using a simple and low-cost hydrothermal calcination method, which was tested as a cathode sulfur carrier for lithium-sulfur batteries. The CC@rGO composite comprises a spherical heterostructure uniformly distributed between graphene sheet layers, preventing stacking the graphene sheet layer. After the introduction of cobalt heterojunction on a graphene substrate, the Co element content increases the reactive sites of the composite and improves its electrochemical properties to some extent. The composite exhibited good cycling performance with an initial discharge capacity of 847.51 mAh/g at 0.5 C and a capacity decay rate of 0.0448% after 500 cycles, which also kept 452.91 mAh/g at 1 C and in the rate test from 3 C back to 0.1 C maintained 993.27 mAh/g. This article provides insight into the design of cathode materials for lithium-sulfur batteries.

## 1. Introduction

Lithium-ion batteries have been studied extremely extensively over the past few decades and are used in portable and mobile electronic devices [1,2]. However, the theoretical energy density of 300 Wh/kg cannot meet the requirements of the growing new energy storage field. Therefore, lithium-sulfur batteries are considered one of the most competitive energy storage devices for the next generation due to their high theoretical specific capacity (1675 mAh/g), energy density (2600 Wh/kg), and the significant advantages of their active substance sulfur such as non-toxicity, low cost and wide source [3]. Despite the above advantages, lithium-sulfur batteries are still hampered by the intrinsic disadvantages of sulfur in the application process. The sulfur’s extremely poor intrinsic conductivity, a large volume change rate during charging and discharging, and the “shuttle effect” caused by soluble polysulfides all cause unstable electrode structure, short cycle life, and low Coulomb efficiency [4,5,6].

Various strategies have been proposed to overcome these challenges, including well-designed cathodes, modified separators, and new-developed electrolytes [7]. In the cathode material design, the use of conductive carbon materials, the design of nanostructured sulfur cathodes, and the incorporation of polar metal materials are the most common and widely used methods. Conductive carbon materials, such as carbon nanotubes [8,9,10,11,12] and graphene [12,13,14,15,16], can encapsulate polysulfides in the internal pores by physical adsorption [17]. However, the interaction between non-polar carbon materials and polar polysulfides is weak and cannot inhibit the diffusion of polysulfides in the long cycle process [18,19]. Although heteroatom doping can effectively polarize the surface of carbon materials [20,21,22], the concentration of existing doping methods is too low to play a role in sulfur fixation. As for polar materials such as metal oxides, they can effectively trap and transform polysulfides due to the strong bonding between them and sulfur, but there are disadvantages such as their poor electrical conductivity and too-strong bonding with polysulfides [19,23,24]. In contrast, polar materials such as metals, metal sulfides, and metal phosphides have high conductivity, however, they have poor adsorption capacity for polysulfides compared to metal oxides [25,26,27], which limits the conversion of polysulfides for multiple uses. Therefore combining metal oxides and metal sulfides and forming heterojunctions is a promising strategy [28]. Different strategies for synthesizing heterojunctions have been continuously proposed, including SnO_2_-SnS_2_ [29], Mn_3_O_4_-MnS [30], and Co_3_O_4_-CoP [31]. For instance, Wang et al. synthesized SnO_2_-SnS_2_ nanosheet heterojunctions to determine for the first time the interfacial effect in lithium-sulfur batteries, which improved the diffusion efficiency of ions and significantly accelerated the redox reaction [29]. Qin et al. designed metal–metal three-layer hollow spheres as sulfur carriers and utilized the separated spatial constraints of the hollow multishell structure to fully utilize the active sites and the built-in electric field, and the assembled cells had remarkable cycling performance and rate performance [30]. Zhang et al. synthesized CC on carbon nanotubes, combining the strong adsorption of oxides with the conversion of phosphides to prepare a composite material with superior electrochemical properties [31]. Most of these studies focus on the structural design and material selection of heterojunctions, while simple and low-cost preparation of heterojunction–carbon materials has been rarely investigated. Therefore, it is instructive to develop heterojunction-carbon materials with reasonable structures and study them for the development of cathode materials for lithium-sulfur batteries.

This study presents a hydrothermal–calcination method for synthesizing rGO-CoO/Co_9_S_8_ heterojunction composite nanomaterials (CC@rGO). CoSO_4_, graphene, and chitosan were used as the Co source, carbon source, and supplementary carbon source, respectively. The Co^2+^ ions were assembled with negatively charged functional groups on graphene oxide through electrostatic adsorption, followed by a hydrothermal-high temperature reduction to generate CoO/Co_9_S_8_ heterojunctions uniformly distributed between graphene lamellae. The CC@rGO electrode exhibited excellent cycling stability and rate performance, thanks to graphene’s large surface area and numerous ion-electron transport channels, the built-in accelerating electric field of CoO/Co_9_S_8_, and the ability to trap and transform polysulfides. This study provides valuable insights into the potential application of carbon material–metal compound composites in lithium-sulfur battery cathode materials, which may lead to the development of more efficient and sustainable energy storage systems.

## 2. Experimental

### 2.1. Preparation of GO

Graphene oxide was prepared by modified Hummer’s method. Two grams g scaled graphite powder were added to 250 mL of a mixed acid solution of sulfuric acid-nitric acid (volume 9:2). A total of 12 g of potassium permanganate was added to it and the temperature was increased to 50 °C (40 min), 60 °C (7 h), 90 °C (30 min), and finally, 30 mL of H_2_O_2_ was added to it to obtain a bright yellow graphene oxide solution. After being cooled, the graphene oxide was left to separate, centrifuged to a pH of 7, and finally freeze-dried, as previously reported by our subject group [32,33].

### 2.2. Preparation of Co_9_S_8_/CoO/rGO

A suspension of graphene oxide was prepared (1.5 mg/mL, solution A), then 2 g of cobalt sulfate and 0.3 g of chitosan were added to 50 mL of deionized water (solution B), and A and B were homogeneously mixed, and transferred to a PTFE-lined reactor, hydrothermally heated at 180 °C for 12 h. The precursor powder was obtained by freeze-drying. The black powder (Co_9_S_8_/CoO/rGO, abbreviated as CC@rGO) was obtained by calcination at 500 °C for 2 h in the Ar atmosphere. rGO samples were made by the same procedure without the addition of cobalt sulfate and chitosan.

### 2.3. Preparation of Battery Cathode

The monomeric sulfur was mixed with the sample at a mass ratio of 7:3 and then molten at 155 °C for 12 h. The active material, conductive agent (Super-P), and binder (PVDF) were ground at 7:2:1 and added to a certain amount of NMP, and stirred for 12 h to make a slurry, coated on carbon-coated aluminum foil, dried at 60 °C for 12 h, and then stamped into a 12 mm × 12 mm circular positive electrode by a press.

### 2.4. Material Characterizations

The microscopic morphology of the samples was observed by scanning electron microscopy (SEM, TESCAN MIRA LMS, Brno, Czech Republic) as well as elemental analysis (EDS, TESCAN MIRA LMS, Czech Republic). X-ray diffraction analysis (XRD, ultima IV, Rigaku, Japan) was used to characterize the sample lattice structure in the interval 10°–90° at 5° per minute. Transmission electron microscopy images (TEM, FEI Tecnai G2F 20, Hillsboro, OR, USA) were used to obtain the internal microstructure. X-ray photoelectron spectroscopy images were acquired by K-Alpha (XPS, Thermo Scientific, Waltham, MA, USA). To obtain Raman spectra, a Renishaw micro confocal laser Raman spectrometer (633 nm) by HR800 (Raman, HORIBA JobinYvon, Palaiseau, France) was used. Thermal gravimetric analysis (TG, TA TGA 550, New Castle, DE, USA) was used to analyze sample sulfur loading. The specific surface area and pore size distribution before and after modification were analyzed using N_2_ adsorption and desorption experiments (BET, Micromeritics 3FLEX, Norcross, GA, USA).

### 2.5. Electrochemical Measurements

A coin cell (type CR2032) was used for electrochemical testing of the rGO as well as the CC@rGO. A 12 mm × 12 mm circular positive electrode sheet was assembled into a cell in a glove box, and the electrolyte used consisted of 1.0 M LiTFSI -DOL: DME with a 1:1 volume ratio and 2% LiNO_3_ (dodochemicals.com, accessed on 11 November 2022). Lithium sheets (Φ15.6 mm, 0.45 mm, China Energy Lithium Co., Tianjin, China) were used for the negative electrode of the half-cells. Coin cell constant current charge/discharge test was performed in the voltage window range of 1.7–2.8 V. Electrochemical AC impedance (EIS) testing, as well as cyclic voltammetry (CV) testing was performed at the Princeton Electrochemical Workstation (VersaSTAT3, Oak Ridge, TN, USA), with EIS frequencies ranging from 1 × 10^−2^ Hz to 10^6^ Hz. Li_2_S_6_ adsorption experiments were used to verify the adsorption capacity of the material (0.05 M Li_2_S_6_ dissolved in 1:1 DME: DOL).

## 3. Results and Discussion

**Figure 1:**
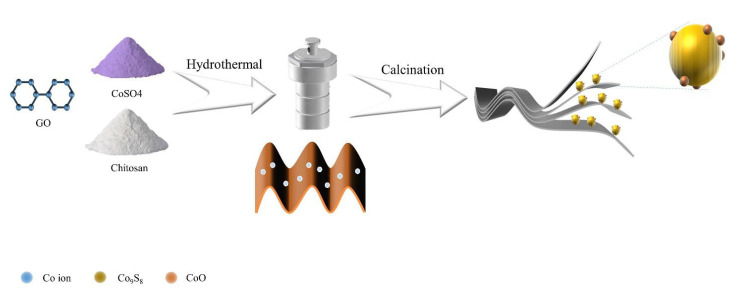
Flow chart of CC@rGO synthesis.

As shown in Figure 1, Co^2+^ combines with the negatively charged oxygen-containing functional group on GO under the effect of electrostatic adsorption, and after the removal of the oxygen-containing functional group by hydrothermal heat, Co^2+^ fills the oxygen vacancies generated after the removal of the oxygen-containing functional group and achieves the uniform distribution of Co^2+^ among the graphene sheets. After calcination at 500 °C, CoSO_4_ was calcined and reduced to CoO and Co_9_S_8_ heterogeneous spheres, which were uniformly distributed between the graphene lamellae, forming a typical wrapping structure. Graphene and heterojunction (composed of cobalt oxide and nine cobalt octa sulfide) composites can not only physically limit the dissolution of polysulfides into the electrolyte through the porous structure of graphene, but also trap and transform polysulfides through the heterojunction, inhibiting the shuttle effect that occurs when polysulfides shuttle through the diaphragm to the surface of the lithium sheet and react to cause a decrease in capacity and lifetime.

To prepare CoO/Co_9_S_8_@rGO composites, CoSO_4_/C@GO precursors were prepared by hydrothermal mixing and calcined to generate CoO/Co_9_S_8_@rGO composites (labeled as CC@rGO).

**Figure 2:**
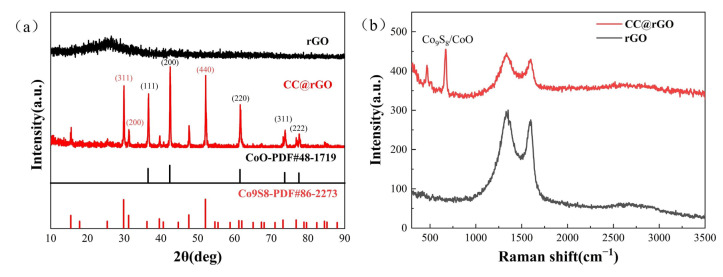
XRD (**a**) and Raman plots (**b**) of rGO and CC@rGO.

The compositional analysis of rGO and CC@rGO was carried out using XRD, as shown in Figure 2a. The diffraction peaks of CC@rGO at 36.49°, 42.38°, 61.49°, 73.67°, and 77.53° mainly correspond to (111), (200), (220), (311), (222) crystallographic planes, respectively, with the standard card PDF#48-1719 matches (CoO), while the diffraction peaks at 29.38°, 31.29°, 52.09° correspond to (311), (200), (440) of Co_9_S_8_, respectively, proving the generation of CoO and Co_9_S_8_. rGO exhibits a distinct (002) interface at 26°, which has the amorphous broad peak that is amorphous carbon at 26°. The composites, on the other hand, did not show a clear broad peak at 26°, which indicates a clear amorphization trend of graphene during the carbon thermal reduction process and the disappearance of the broad peak [34].

Raman spectroscopy was performed for CC@rGO and rGO to measure the disorder of different samples, and the results are shown in Figure 2b. The two broad bands of CC@rGO appear at 1359 cm^−1^ and 1600 cm^−1^, corresponding to the D and G peaks [12,35], respectively, while the D and G peaks of CC@rGO are shifted to the right at 1335 cm^−1^ and 1590 cm^−1^, respectively, which was believed to be due to the addition of heterojunctions that make the material structure vibrate and shift. The double peaks of CC@rGO at 466 cm^−1^ and 676 cm^−1^ were considered to be strong interactions between CoO and Co9S8 [36,37]. Compared to rGO, the I_D_/I_G_ of CC@rGO is reduced from 1.1 to 1.035 and the defects of the material are reduced [38]. The addition of Co oxide particles, encapsulated by graphene, decreases the D peak, which is caused by the lamellar encapsulation property of graphene on transition metals, and the D peak includes oxygen-containing functional groups and defects in the material itself [39]. Meanwhile, the 2D peaks of CC@rGO did not change significantly compared to the 2D peaks of rGO, indicating that the incorporation of heterojunctions did not lead to the stacking of graphene lamellae.

**Figure 3:**
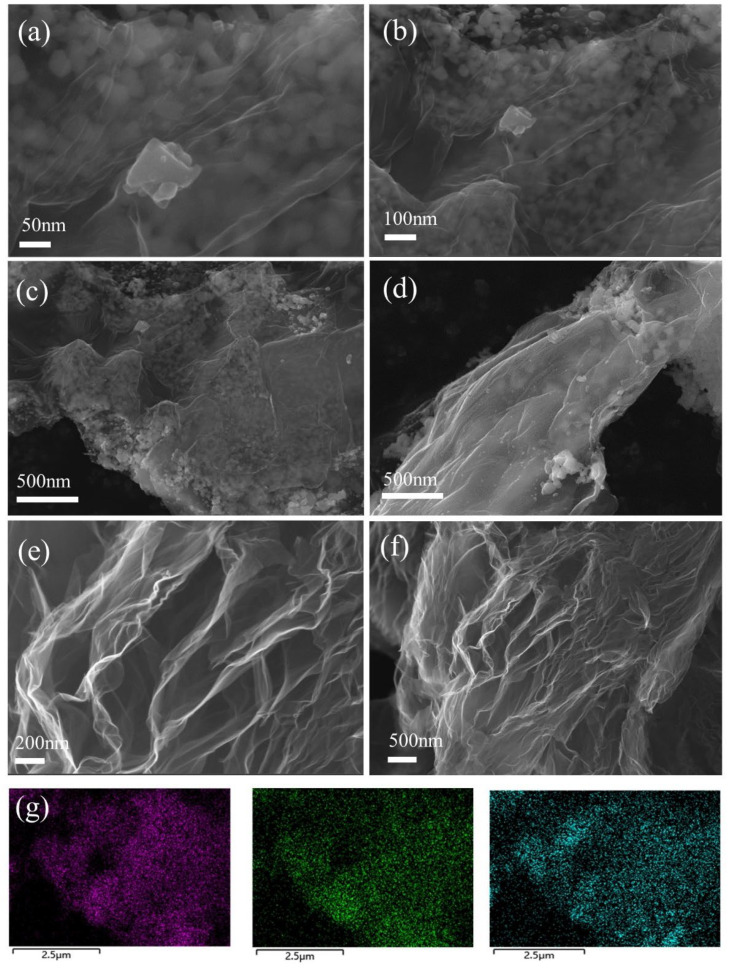
SEM images of CC@rGO (**a**–**d**) and rGO (**e**,**f**) and elemental energy spectrum of Co, O, S (**g**).

The microstructure and elemental distribution of rGO and CC@rGO were observed by scanning electron microscopy and elemental energy spectroscopy, respectively. As shown in Figure 3e,f, rGO shows a distinct muslin lamellar shape after hydrothermal calcination, which is consistent with the morphology of reduced graphene oxide prepared in the literature, while irregular heterogeneous ellipses or squares formed by CoO and Co_9_S_8_ have uniformly distributed between graphene lamellar layers as seen in Figure 3a–d. This helps to suppress the stacking of graphene lamellar layers due to van der Waals forces, increase ion and electron transport channels, and enhance the sulfur-carrying capacity. Meanwhile, the large specific surface area of rGO provides abundant space for sulfur loading, which can give full play to the adsorption and conversion ability of polysulfides by heterojunctions and effectively improve the electrical conductivity of the composites, and form sulfur cathode composites with stable structures. An elemental analysis of CC@rGO is shown in Figure 3g, which further demonstrates the uniform distribution of Co, S, and O in the sample. The homogeneous distribution of Co elements also demonstrates the binding and homogeneous distribution of Co^2+^ with oxygen-containing functional groups on graphene during the hydrothermal process.

**Figure 4:**
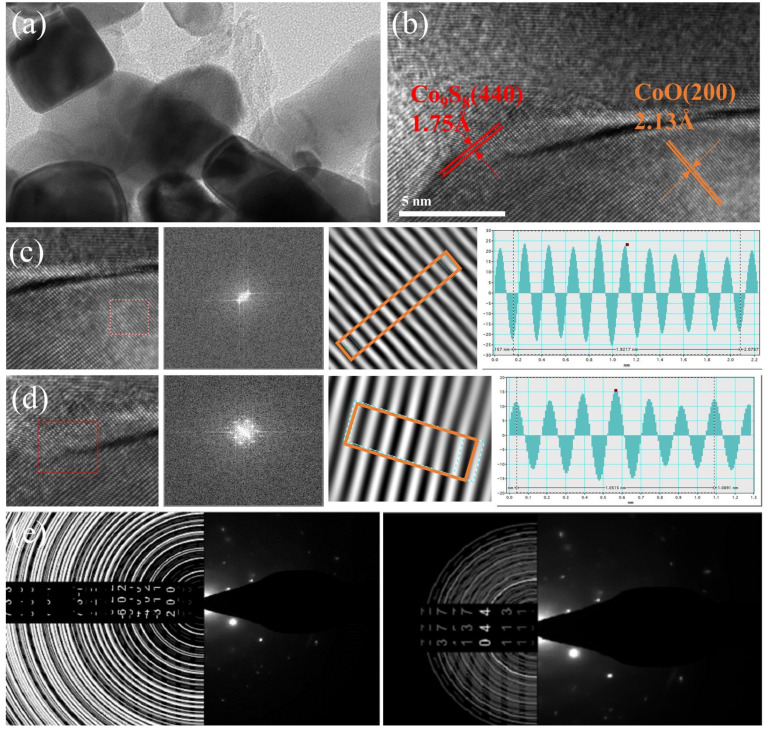
TEM images of CC@rGO (**a**), HRTEM images of CC@rGO (**b**), lattice streak analysis, and SAED diagram of CC@rGO (**c**–**e**).

As shown in Figure 4a,b, the heterojunctions in CC@rGO show irregular ellipses or squares. The 0.213 nm lattice stripes correspond to the (200) crystal plane of CoO, while 0.124 nm and 0.175 nm lattice stripes correspond to the (800) and (440) crystal planes of Co_9_S_8_, respectively (Figure 4c–e). This is consistent with the XRD results, which demonstrate the generation of CoO and Co_9_S_8_ and the presence of heterogeneous interfaces. Due to the interaction between CoO and Co_9_S_8_, abundant inhomogeneous interfaces are formed between their particles, and the built-in electric field induced through the heterogeneous interfaces helps ions migrate in the lithium-sulfur cell, while graphene as the substrate provides abundant transport channels and provide support for good electrochemical performance.

**Figure 5:**
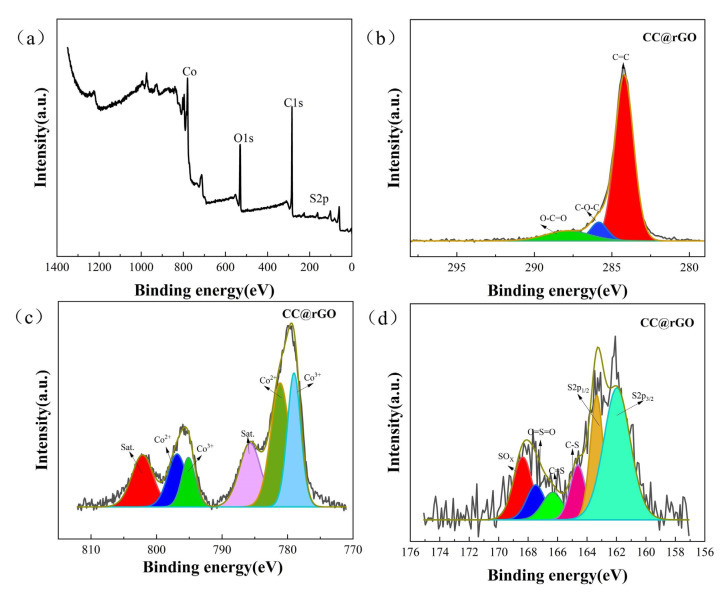
(**a**) Elemental spectrum, (**b**) C spectra, (**c**) Co spectra, (**d**) S spectra of CC@rGO.

The chemical bonding of the composites was analyzed by XPS. As shown in Figure 5c, an XPS analysis of CC@rGO samples existed for C, O, S, and Co. Figure 5d shows the high-resolution peak fitting curves for C1s, having 284.2 eV, 285.58 eV, and 287.83 eV corresponding to the C=C bond, C-O-C bond, and O-C=O bond, respectively [40,41]. Referring to the high-resolution XPS spectra of Co2p shown, the two peaks at 779 and 795 eV correspond to 2p_3/2_ and 2p_1/2_ of Co^3+^, the peaks at 781.2 and 797 eV correspond to 2p_3/2_ and 2p_1/2_ of Co^2+^, while the two peaks at 785.8 and 802 eV correspond to the satellite peaks of Co [35,42]. 

There are also two sets of satellite peaks (802.08 eV, 785.83 eV) in the S2p high-resolution spectrum, with peaks at S2p_3/2_ (161.8 eV) and S2p_1/2_ (162.8 eV) attributed to metal-S bonds [35,43]. The two peaks at 163.7 and 165.3 eV were attributed to C-S-C bonding and C-S bonding, respectively. The remaining peaks at 168.8 and 170 eV represent the C-SO_x_-C species [16,41,44]. The multivalent form of Co aids in accelerating the LiPSs power conversion process during battery discharge.

**Figure 6:**
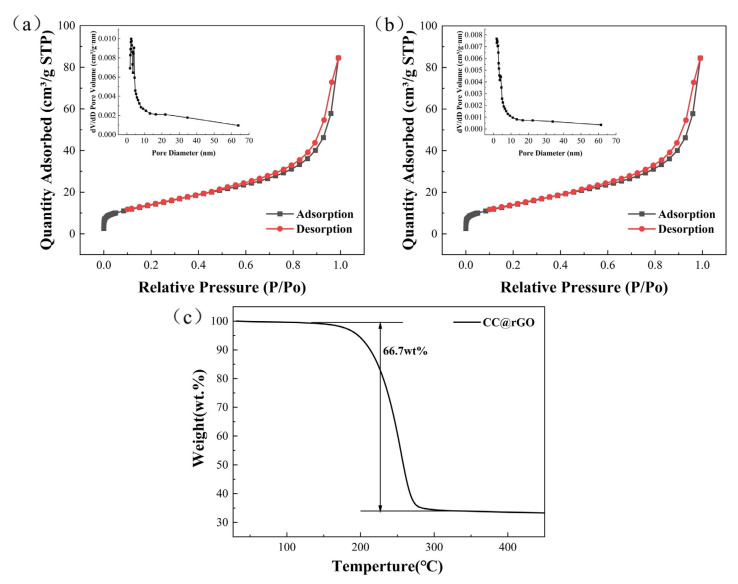
Desorption curve and pore size distribution of (**a**) rGO (**b**) CC@rGO, (**c**) TG of CC@rGO.

As shown in Figure 6, both rGO and CC@rGO exhibit type IV desorption curves [45,46], indicating that both are mesoporous materials and in sheet form. Compared with rGO, the CC@rGO specific surface area decreased from 49.73 m^2^/g to 46.112 m^2^/g after compounding the heterojunctions due to the incorporation of heterogeneous spheres with a smaller specific surface area. The pore size (calculation: 4 V/A by BET), on the other hand, decreased from 8.16 nm to 4.46 nm. The smaller pore size will melt the sulfur-loaded process to encapsulate the sulfur well in the pore channel and maintain sufficient stability. At the same time, during the melting process, due to the good wettability between carbon and sulfur, sulfur will penetrate inward through the capillary of carbon, and when the pore size becomes larger, the capillary force is not enough to let sulfur enter completely. In this way, the small pore size will achieve a better encapsulation of sulfur as well as a better inhibition of polysulfide diffusion. In addition, the small pore size will make the sulfur layer on the material surface thinner, improve the sulfur utilization, and accelerate the electron and ion transfer efficiency; meanwhile, increasing the contact area of the electrolyte will promote the electrolyte to infiltrate the material, reduce the interfacial impedance, and finally reduce the electrochemical polarization [47,48,49].

The composites’ large surface area and thin mesoporous structure will make it easier for the cell’s electrolyte to penetrate the material, which will lower interface impedance, increase charge exchange efficiency, hasten the conversion of sulfur intermediate species, and enhance the cell’s electrochemical performance. As shown in Figure 6c, the thermogravimetric curves of CC@rGO in argon atmosphere, the sample starts to lose weight around 160 °C and stops losing weight around 280 °C. The results indicate a sulfur content of 66.7%, which is in general agreement with the sulfur loading of the experimental part.

**Figure 7:**
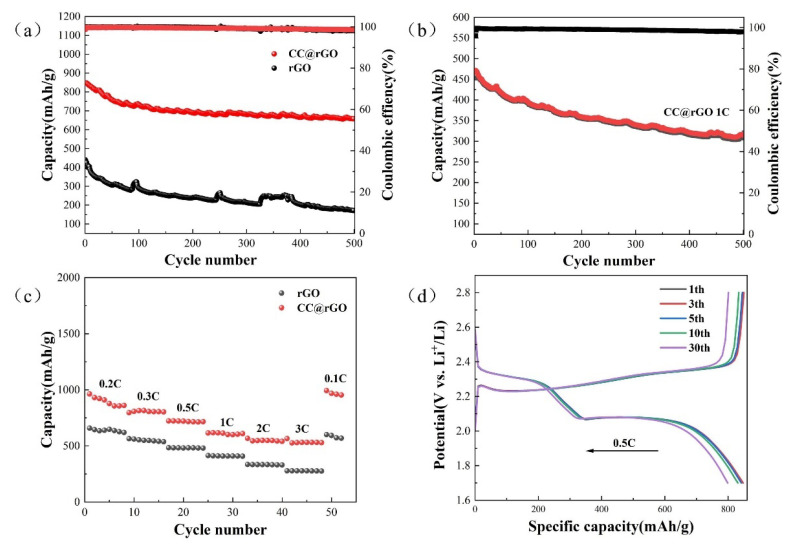
(**a**) 0.5 C cycle plots of CC@rGO and rGO, (**b**) 1 C cycle plots of CC@rGO, (**c**) rate performance, (**d**) charge/discharge curves of CC@rGO.

To characterize the electrochemical performance of the composites with wrapping structure, a series of electrochemical characterizations were performed for the cells assembled with rGO and CC@rGO, respectively. To characterize the electrochemical properties of the composites, CC@rGO and rGO were assembled into CR2032 button cells, respectively. As shown in Figure 7a, compared with rGO with 437.81 mAh/g in the first cycle, CC@rGO has a high discharge capacity of 847.51 mAh/g in the first cycle, and the decay rate is only 0.0448% per cycle after 500 cycles, which is much lower than that of rGO; this indicates that CC@rGO has effectively improved the conductivity and structural stability of the material after the introduction of CoO/Co_9_S_8_ heterostructure. It can effectively activate more sulfur-active material while maintaining good cycling stability and promoting the kinetic conversion of polysulfides. The cycling test of CC@rGO using 1C cycling current shows that the first turn discharge capacity is 452.91 mAh/g and the decay rate is 0.0639% after 500 cycles, which further demonstrates the good structural stability and electrical conductivity of the composite material.

The rate electrochemical characterization of CC@rGO was performed and the results are shown in Figure 7c. In the button cell, the discharge capacities of rGO and CC@rGO in 0.2 C, 0.3 C, 0.5 C, 1 C, 2 C, 3 C, and 0.1 C were 657.79 mAh/g, 564.07 mAh/g, 483.37 mAh/g, 413.28 mAh/g, 334.37 mAh/g, 277.64 mAh/g, 600.13 mAh/g and 962.96 mAh/g, 807.01 mAh/g, 722.08 mAh/g, 614.81 mAh/g, 568.04 mAh/g, 529.53 mAh/g, and 993.27 mAh/g, respectively. The rate performance proves that CC@rGO has good multiplicative properties, which further supports the structural stability and cycling performance of the composite under high currents. The cyclic charge–discharge plateau curves in Figure 7d shows that the lithium-sulfur battery prepared by CC@rGO exhibits a typical dual charge–discharge voltage plateau (phase I, 2.4–2.1 V; phase II, 2.1–1.7 V) [13]. After 30 cycles, the typical charge–discharge plateau can still be maintained and the △E is unchanged, which indicates that CC@rGO as a sulfur carrier has good electrode structural stability and electrode kinetic performance, corroborating that the composite material can achieve excellent long-cycle performance. In terms of electrochemical performance, the introduction of polar CoO/Co_9_S_8_ heterojunctions between nonpolar graphene sheets provides abundant polysulfide action sites, effectively suppressing the loss of active material sulfur caused by the “shuttle effect” and reducing the appearance of dead sulfur during the cycling of lithium-sulfur batteries.

**Figure 8:**
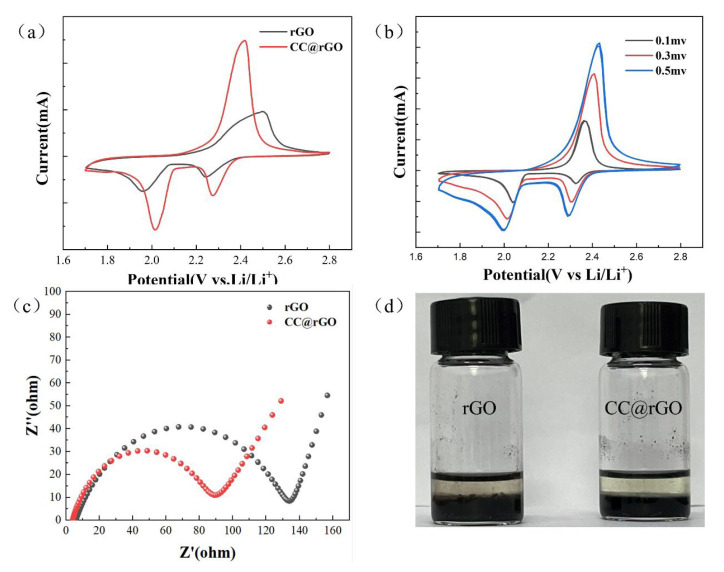
(**a**) CV plots of CC@rGO and rGO at 0.1 mv/s sweep speed, (**b**) CV plots of CC@rGO at different sweep speeds, (**c**) EIS plots of CC@rGO and rGO, (**d**) Li_2_S_6_ adsorption test chart.

The electrochemical performance of rGO and CC@rGO cells was compared at a scan rate of 0.1 mv/s and a voltage window of 1.7–2.8 V. The two typical reduction peaks (2.25 V, 2.01 V) characterized by CC@rGO correspond to the reduction of S to soluble polysulfide (Li_2_S_x_, 4 ≤ x ≤ 8) and the reduction of soluble polysulfide to insoluble polysulfide (Li_2_S_2_, Li_2_S) during discharge, respectively [50]. Compared with rGO, the CC@rGO has a smaller electrochemical polarization with a higher peak after the introduction of the heterostructure, implying that the composite has better kinetic catalytic performance as well as charge and discharge capacity [51]. This indicates that the introduction of the heterojunctions results in a stronger catalytic effect and faster reaction kinetics of the cell [52]. In Figure 8b, the electrochemical performance of the CC@rGO cell is investigated at different sweep rates, and the cell polarization increases with increasing sweep rate, but the curve shape does not change much and the multi-turn curves at uniform sweep rates are in good agreement, which proves that CC@rGO has good structural stability and is consistent with the results of charge/discharge plateau curve and rate curve.

The electrical conductivity of the cells assembled from the composites was further tested, and the Nyquist curves were shown in Figure 8c. Compared with rGO, the R_ct_ (charge transfer resistance) of CC@rGO is reduced from 133 Ω to 88 Ω. The improved tilt of the Warburg curve in the low-frequency region is attributed to the increased conductivity of the composite material after the addition of the heterostructure, which can better perform the adsorption-transformation of polysulfides [7]; meanwhile, the electrostatically adsorbed heterojunction effectively prevents the stacking of graphene and increases the reactive sites as well as ion transport channels, which enhances the ion transfer diffusion in the cell. 

As shown in Figure 8d, Li_2_S_6_ adsorption experiments were carried out to further verify the adsorption capacity of the composites. After 12 h adsorption in 0.05 M Li_2_S_6_ solution, the supernatant of CC@rGO on the right side was clarified, while the rGO on the left side still showed a pale yellow color, indicating that CC@rGO has excellent static adsorption ability, which is consistent with the previous characterization results. This indicates that the composites have a better ability to trap polysulfides, which is attributed to the excellent adsorption of polysulfides by metal oxides and the porous structure of graphene, both of which synergistically enhance the performance of the composites.

In Table 1, the work is compared with the previous graphene-based composite lithium-sulfur battery cathode, and although the initial discharge capacity is slightly lower at 847.51 mAh/g, the material shows excellent capacity retention over long cycles, maintaining a high capacity retention and low decay rate (0.0448%) over 500 cycles, demonstrating that the material has good structural stability and can achieve longer cycle life and better Coulomb efficiency.

## 4. Conclusions

In summary, we propose the preparation of CoO/Co_9_S_8_ heterojunction-rGO composites by electrostatic adsorption and calcination. CoO/Co_9_S_8_ heterojunction acts as a polysulfide adsorption transformation, while rGO acts as a sulfur-carrying carbon carrier and provides ion and electron transport channels. Due to the wrapping structure, it can play a synergistic-catalytic role well, suppressing the “shuttle effect” of LiPSs and accelerating the electro-kinetic reaction. The results show that the CC@rGO sulfur cathode has good electrochemical performance, good rate performance, and cycling stability at different current densities, and the Coulomb efficiency is always maintained above 97%. Therefore, this electrode material design can provide new insights into the design and development of sulfur carriers for lithium-sulfur battery cathodes.

## Figures and Tables

**Table 1:** Comparison of the results of this work with previous studies.

Sample	Electrochemical Performance	Decay Rate	
ZIF-8@rGO/S	1544 mAh/g at 0.2 C	0.33% after 200 cycles	[53]
Co_9_S_8_@S/GO	1057 mAh/g at 0.1 C	0.033% after 100 cycles	[54]
TiO_2_ NTs/GO hybrid	850.7 mAh/g at 0.1 C (after 100 cycles)	0.409% after 100 cycles	[55]
Ni_3_(HITP)_2_@GO/S	959.3 mAh/g at 0.5 C	0.1325% after 400 cycles	[56]
NCF-G@S	923.8 mAh/g at 0.5 C	0.212% after 150 cycles	[21]
CC@rGO	847.51 mAh/g at 0.5 C	0.0448% after 500 cycles	This work

## Data Availability

The data presented in this study are available on request from the corresponding author. The data are not publicly available due to personal privacy.
